# Fertility preservation in pediatric healthcare: a review

**DOI:** 10.3389/fendo.2023.1147898

**Published:** 2023-05-03

**Authors:** Lin Chen, Zirui Dong, Xiaoyan Chen

**Affiliations:** ^1^ Reproductive Medical Center, The First Affiliated Hospital, Sun Yat-sen University, Guangzhou, China; ^2^ Department of Obstetrics and Gynecology, The Chinese University of Hong Kong, Hong Kong, Hong Kong SAR, China; ^3^ Shenzhen Research Institute, The Chinese University of Hong Kong, Shenzhen, China; ^4^ Maternal-Fetal Medicine Institute, Shenzhen Baoan Women’s and Children’s Hospital, Shenzhen University, Shenzhen, China; ^5^ The Fertility Preservation Research Center, Department of Obstetrics and Gynecology, The Chinese University of Hong Kong, Hong Kong, Hong Kong SAR, China

**Keywords:** fertility preservation, oocyte cryopreservation, ovarian tissue cryopreservation, testicular tissue cryopreservation, pediatric

## Abstract

Survival rates for children and adolescents diagnosed with malignancy have been steadily increasing due to advances in oncology treatments. These treatments can have a toxic effect on the gonads. Currently, oocyte and sperm cryopreservation are recognized as well-established and successful strategies for fertility preservation for pubertal patients, while the use of gonadotropin-releasing hormone agonists for ovarian protection is controversial. For prepubertal girls, ovarian tissue cryopreservation is the sole option. However, the endocrinological and reproductive outcomes after ovarian tissue transplantation are highly heterogeneous. On the other hand, immature testicular tissue cryopreservation remains the only alternative for prepubertal boys, yet it is still experimental. Although there are several published guidelines for navigating fertility preservation for pediatric and adolescent patients as well as transgender populations, it is still restricted in clinical practice. This review aims to discuss the indications and clinical outcomes of fertility preservation. We also discuss the probably effective and efficient workflow to facilitate fertility preservation.

## Introduction

Long-term survival for children and adolescents diagnosed with malignancy has steadily increased and exceeded 80% over the past decade (1–3). As these cancer survivors reach adulthood, a substantial proportion of them experience infertility associated with previous gonadotoxic chemotherapy and/or radiotherapy (4, 5). In addition to oncology treatment, other non-oncological conditions and related therapy may raise fertility problems, including nephrotic syndrome (6), Turner Syndrome (7) and systemic lupus erythematosus (8). Currently, fertility issues have been increasingly recognized as a major concern for those newly diagnosed patients and their families (9, 10). Failing to achieve parenthood raises tremendous psychosocial stress on patients and their families and impairs their well-being. While scientists have established a range of methods for fertility preservation, including embryo cryopreservation, gamete cryopreservation, and gonad tissue cryopreservation (11), the clinical practice is not as satisfactory as expected (12, 13). This review aims to discuss the indications, methods, and clinical outcomes of fertility preservation in the pediatric and adolescent populations. The potential effective and efficient workflow to facilitate fertility preservation is discussed as well.

## Indications for fertility preservation in the pediatric and adolescent populations

### Oncological causes

The incidence of pediatric and adolescent cancers is estimated to range between 50 to 200 cases per million children per year (14, 15) and more than 80% of these cancers are now potentially curable with current treatments (1, 2). Chemotherapy and radiotherapy in cancer treatments can lead to temporary, long-term and permanent gonadal toxicity, making fertility impairment another issue that distresses cancer survivors and their families (16). Alkylating agents are highly gonadotoxic and are associated with premature ovarian insufficiency (17, 18) and oligo- or azoospermia (19) depending on agent and dose (17, 20).

In females, these treatments substantially accelerate the activation and atresia of primordial follicles, leading to premature ovarian insufficiency (POI) and permanent amenorrhea (18, 21–24). Depending on agents and regimes, Impact on fertility may be broadly classified in low (<20%), medium (20-80%), or high (>80%) (25). A 13-fold increased chance of developing premature menopause was observed in a childhood cancer survivor study (26). Ovarian radiation, on the other hand, can cause 50% of follicle depletion at the dosage of 2 Gy and 60% chances of ovarian insufficiency at 2.5-5 Gy (25). Besides, long-term follow up of pediatric patients also demonstrated significant decline in anti-Müllerian hormone (AMH) after cancer therapy (23, 27), suggesting fertility losses and future fertility problems.

Spermatogenesis is particularly sensitive to chemotherapy and radiotherapy (28). Long-term follow up observed 25% and 28% of adult survivors of childhood cancer suffered from azoospermia and oligospermia after chemotherapy with cyclophosphamide equivalent dose less than 4000 mg/m² (29). In some regimes, several agents were administrated together, when cyclophosphamide is given > 7500 m g/m², almost all patients developed permanent azoospermia (16). Similarly, exposure to radiation causes germ cell loss in a dose-dependent manner (30), with immature spermatogonia the most radiosensitive, followed by spermatocyte and spermatid (28). Radiation at 0.1 Gy can result in morphological and quantitative changes to spermatogonia, increasing the dosage leads to spermatocyte and spermatid reduction (31). The threshold of radiation dose leading to permanent azoospermia remains unclear. But Castillo et al. found that all boys with acute lymphoblastic leukemia receiving testis radiotherapy at dose over 12 Gy developed azoospermia (19). A more recent study suggests that testicular radiation > 6 Gy may lead to permanent infertility (31).

### Non-oncological causes

Younger patients affected by certain non-oncological medical conditions which require gonadotoxic treatments are potential candidates for fertility preservation as well (17, 32, 33). For example, gonadotoxic alkylating agents are widely used for diseases including nephrotic syndrome (6), systemic lupus erythematosus (34, 35), refractory idiopathic thrombocytopenic purpura (36). Also, a range of hematopoietic disorders, including thalassemia major, sickle cell anemia, aplastic anemia and myeloproliferative diseases, may be treated with hematopoietic stem cell transplant, which preconditions alkylating chemotherapy with or without radiotherapy (37, 38). In addition, some diseases can affect patients’ fertility at an early age, including Turner syndrome (7, 39), Klinefelter’s syndrome (40), fragile X syndrome (33), endometriosis (41), and gonad injury (42). Transgender populations receiving gender-affirming treatments may also require fertility preservation (43). Some of the most common non-oncological conditions which may require consideration of fertility preservation is presented in **Table 1**.

**Table 1:** Non-oncological indications for fertility preservation.

Conditions	Diseases
Autoimmune diseases (35, 44)	Systemic lupus erythematosus, Crohn's disease, Behcet’s disease, Sjogren's syndrome, systemic scleroderma, nephrotic syndrome, multiple sclerosis, acute progressive nephritis syndrome, *etc.*
Hematopoietic stem cell transplantation (38)	β-thalassemia major, severe aplastic anemia, sickle-cell disease, Fanconi’s anemia, *etc.*
Other conditions causing POI or spermatogenic failure	Turner syndrome (7), Klinefelter's syndrome (40), fragile X syndrome (33), endometriosis (41), ovarian/testicular torsion, benign ovarian tumors, galactosemia (45), gonad injury (42), *etc.*
Transgender populations (43)	Not applicable

POI, premature ovarian insufficiency.

Potential risks for fertility impairment vary depending on patients’ age, gender, body mass index, medical condition, and subsequent treatment scheme. A comprehensive and individual assessment is essential to determine the appropriate timing and methods for fertility preservation (46). Previous guidelines have extensively discussed the fertility risk assessment of specific agents or therapy regimes (25, 47–51), which have provided useful guidance to current practice.

## Available options for fertility preservation

### Females

#### Oocyte cryopreservation

Embryo cryopreservation, as a long-established fertility preservation method, can guarantee the best outcomes for fertility preservation. However, oocyte cryopreservation is preferred since most adolescents are unlikely to have a permanent partner and using donor sperm is less desired and poses ethical issues (11, 17). Since 2012, oocyte cryopreservation is no longer considered an experimental method for fertility preservation (52). However, outcomes in adolescents are less clear.

**Figure 1:**
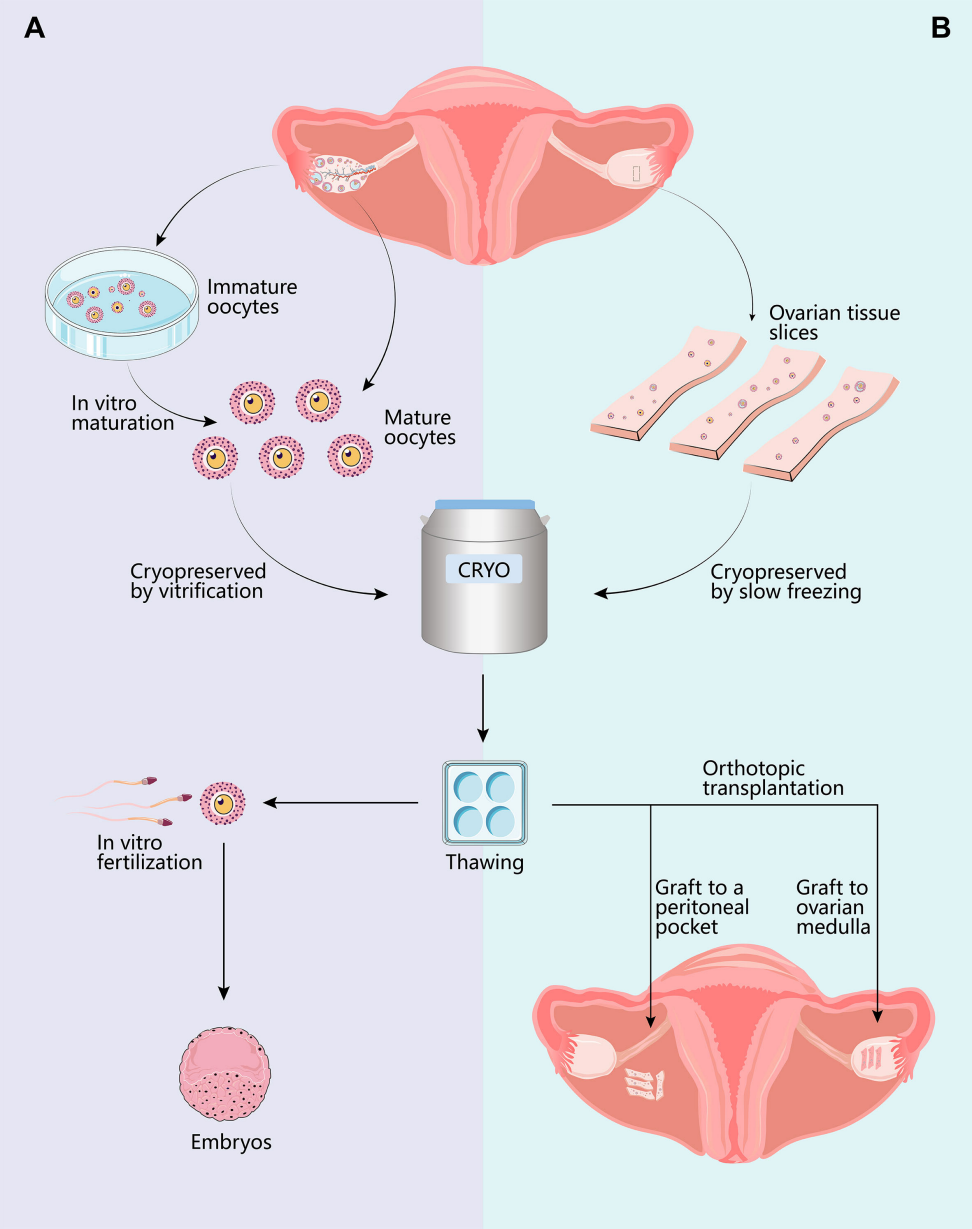
Options for female fertility preservation. For pubertal patients, mature oocyte cryopreservation is the optimal strategy. Controlled ovarian hyperstimulation and oocyte retrieval can be completed within two weeks if the treatments can be delayed. Another method requiring less time for ovarian stimulation is cryopreservation of *in vitro* matured immature cumulus-oocyte-complex (COCs). Additionally, immature COCs can be obtained while harvesting ovarian tissue for cryopreservation. Thawed oocytes are utilized for *in vitro* fertilization with intracellular sperm injection **(A)**, resulting in live birth rates per transfer varying between 39% and 52%. If the patient is prepubertal or requires immediate treatments, ovarian tissue cryopreservation remains the only option. The ovarian cortex is surgically removed, dissected, and cryopreserved. While vitrification is ideal for the cryopreservation of oocytes, slow-freezing is currently preferred for the preservation of ovarian tissue. Thawed ovarian slices may be transplanted either orthotopically or heterotopically **(B)**. Transplantation to orthotopic sites (broad ligament and ovarian medulla) provides the chance for spontaneous conception, whereas transplanting to heterotopic sites necessitates assisted reproductive techniques. The overall live birth rates after OTT range from 18.2% to 43.3%.

Controlled ovarian stimulation is the most effective strategy to obtain mature oocytes (53). However, conducting ovarian stimulation on a basis of diagnosed disease requires modifications to conventional protocols to address potential restrictions, including limited time allowed, and temporary exposure to high estradiol levels (54). Advances in ovarian stimulation have allowed fertility specialists to finish ovarian stimulation and oocyte retrieval within two weeks (55, 56). In urgent situations, the gonadotrophin-releasing hormone (GnRH) antagonist protocol is considered optimal for its short time and safety. Meanwhile, random and double stimulation are feasible alternatives (32, 57). In non-urgent situations, on the other hand, both GnRH antagonist protocol and long protocol are appropriate (32). Anti-estrogenic agents may be added to abolish estradiol reproduction in estradiol-sensitive diseases (53, 54). In addition, cryopreservation of *in vitro* maturated oocytes may be a feasible strategy when present with time constraints (58), which eliminates potential estrogen elevation and minimizes delay in treatment. Immature oocytes can be obtained at the time of ovarian tissue cryopreservation or oophorectomy as well (59). Various cryopreservation methods have been developed to freeze oocytes. If patients survive the original diseases and desire pregnancy in the future, these oocytes can be thawed and used for assisted reproductive techniques (**Figure 1A**). Recent advances in cryoprotectants, cryopreservation techniques (vitrification), and fertilization with intracytoplasmic sperm injection (ICSI) have significantly improved the clinical efficacy of cryopreserved oocytes (60–62). A series of studies which investigated the efficacy of different cryopreservation protocols concluded that vitrification outperforms slow freezing (63). The survival rate of vitrified oocytes ranges between 73.6% and 92.7%, significantly higher than that of slow freezing (58.0%-72.3%) (63–70). Vitrification is also superior regarding other outcomes like fertilization, implantation, clinical pregnancy, and live birth (63, 65, 71).

#### GnRH-agonist protection

The clinical efficacy of gonadotrophin-releasing hormone agonists (GnRH-a) during chemotherapy is controversial (54) and current recommendations regarding its use remain conflicting (11, 32, 53). Some meta-analyses evaluated the protective effect of GnRH-a during chemotherapy in premenopausal patients with breast cancer or lymphoma. Lower risks of chemotherapy-induced POI/amenorrhea and a higher number of spontaneous pregnancies after GnRH-a withdrawal were observed in the study group (72–75). However, the evidence is relatively weak due to heterogeneous populations, varying chemotherapy regimens and study endpoints (72, 76, 77). More importantly, these studies were conducted in adult subjects with an already established HPO axis. Relevant studies among adolescent patients with cancer are scarce (78, 79). A prospective study found GnRH-a administration during chemotherapy protected ovarian function and preserved fertility in adolescent patients (78). Another retrospective study drew a similar conclusion, with normal ovarian function maintained at the clinical, laboratory, and ultrasonic levels in 27/36 patients after GnRH-a co-administration (79). Overall, well-designed, large, prospective, randomized, controlled trials are essential to determine the protective effect of GnRH-a in children and adolescent patients (80).

#### Ovarian tissue cryopreservation

Ovarian tissue cryopreservation (OTC) is perhaps the sole option for fertility preservation in prepubertal children and post-pubertal adolescents who cannot delay the start of chemotherapy (11, 17, 32, 53, 81). Roughly 50% of the cortex from one ovary is surgically removed, dissected, and cryopreserved for future use (82, 83). The ovarian tissue is cryopreserved by either slow freezing (84–86) or vitrification (87). When a patient intends to restore ovarian function and/or fertility, the cryopreserved tissue can be thawed and replaced (**Figure 1B**).

In 1994, Gosden et al. successfully restored ovarian function to several castrated sheep using frozen-thawed ovarian slices (88). Symbolically, this has resulted in several lambs and maintained long-term ovarian function for up to 2 years (89). After that, studies of cryopreserved human ovarian tissue reported normal follicular morphology after thawing (90), follicular survival (91), and growth of follicles to antral stages (92) when replaced to immunodeficient mice. Thereafter, follicular growth (93, 94), ovarian endocrine function restoration (94), and *in vitro* embryo formation (95) were reported after being transplanted to humans. The first live birth after autologous ovarian tissue transplantation (OTT) using cryopreserved ovarian tissue in humans was documented in 2004 (96) and the second in 2005 (97). Since the milestone event, ovarian tissue cryopreservation and transplantation is gaining increasing attention in the field of fertility preservation. The cumulation of success has recently made it an accepted technique for fertility preservation (53).

Multiple transplantation strategies have been developed. The frozen ovarian tissue can be replaced either orthotopically (17) and/or heterotopically (98). Orthotopic sites include the remaining ovary (96) and peritoneal pockets created on the broad ligament (99). The orthotopic graft provides the possibility of spontaneous pregnancy because of the proximity to the fallopian tube (98, 100–103). Notably, the first live birth was conceived spontaneously without either ovarian stimulation or *in vitro* fertilization (IVF) (96). In some studies, more pregnancies and live births were obtained naturally (99, 101, 102, 104). Many women have been reported to conceive and deliver more than once (104), with 3 cases delivering three times (105, 106) and one case conceiving four times (107). Heterotopic sites include subcutaneous areas in the forearm (98), abdomen wall (95), chest wall (100), breast (108), rectus muscle (108), and subperitoneal tissue (109), where a favorable environment for follicular development such as optimal temperature, paracrine factors, and blood supply may not be provided (100, 108). Thus, the procedure is adopted less frequently (110). Heterotopic autografting eliminates the possibility of conceiving naturally but not with the use of assisted reproductive techniques (ART). Clinical pregnancy (110) and live birth (111) following this procedure have been reported recently, partly removing its controversies. Meanwhile, the procedure also offers several potential advantages (100, 112), including, (1) less invasive surgery; (2) easier follicular monitoring and oocyte retrieval for IVF; (3) easier monitoring for cancer recurrence and removal of the graft, if necessary; and (4) more cost-effective options in case of repetitive transplantations. For some females who wish to restore ovarian function but do not desire pregnancy (98, 99), these advantages probably make heterotopic autograft a potentially preferred option.

Survival of grafted tissue and ovarian follicles depends on several factors, including the timing and location of transplantation, surgical techniques, and most importantly, the levels of revascularization soon after the procedure (113). Studies suggest that it takes 48 hours to revascularize after OTT in rodents (114, 115) but it may take up to 5 days in humans (115). In addition, research shows most follicles die before complete revascularization, with more than 70% of primordial follicles failing to survive the procedure in both humans (116) and sheep (89). There are challenges to further improving the survival of the graft and clinical outcomes (54, 113). On the other hand, cryopreserved ovarian tissue can be transplanted repeatedly in case of replantation failure (98). In a review including 318 women and 369 OTTs worldwide, Gellert et al. found that the average amount of transplanted tissue at the first OTT accounted for 46%, with 37% and 38% of the total amount of cryopreserved tissue being transplanted for the second and the third time, respectively (102). It seems a feasible strategy to extend the duration of ovarian function by repeating grafting procedures.

One of the leading concerns over the autograft of cryopreserved ovarian tissue is the risk of reintroducing malignant cells among malignancy survivors (100), which is considered high in hematological malignancies like leukemia and Burkitt lymphoma, and moderate in the case of Ewing sarcoma, advanced breast cancer, colon cancer, cervical adenocarcinoma (54, 112). In a recent systematic review, metastases were repeatedly detected in ovarian tissue obtained from patients with leukemia, but it was less common in other malignancies (117). Several methods have been applied to detect possible malignancy contamination before transplantation, such as histology (118), immunohistochemistry (119), and polymerase chain reaction (if specific markers are available) (117, 120). It has been proposed that ovarian tissue might be first xenografted to immunodeficient mice to assess the risk before grafted to humans (121). The recurrence rate after ovarian tissue graft in several large cohorts ranges between 3.9% and 7.0% (98, 99, 102), with a study comparing the relapse rate with those who did not accept transplantation and demonstrating similar recurrence rate (7%, 3/41 vs. 7%, 48/691) (99). None of these malignancy relapses was deemed related to OTT but dependent on the primary disease (98, 102), which has been endorsed by multiple studies (98, 102, 103, 107, 111, 122–126). Nonetheless, further studies are warranted to determine the safety of autograft of ovarian tissue among malignancy survivors (100, 102, 119, 121).

### Males

#### Sperm cryopreservation

Sperm cryopreservation with masturbation is the easiest and most reliable method for fertility preservation for pubertal boys (11, 20, 33, 127, 128). Penile vibro-stimulation, as a noninvasive method, can be an alternative when having difficulties with masturbation (20). However, considering the invasiveness, electro-ejaculation and testicular sperm extraction (TESE) should be conducted only after weighing the benefits and harms (20, 129).

#### Cryopreservation of immature testicular tissue

Cryopreservation of immature testicular tissue (ITT) is the only fertility preservation option for prepubertal boys as spermatogenesis is absent (11). Small pieces of immature testicular tissue are surgically removed for cryopreservation. Yet still experimental, it is stressed that the procedure is provided exclusively for research purposes under ethical approval or novel technologies governance (20, 33, 76, 130). According to a survey, at least 1033 prepubertal boys aged between 3 months and 18 years have received the procedure (131). Multiple surveys reveal parents are willing to embrace the experimental technique (132–135), in hope that future advances in reproductive techniques will allow fertility restoration by the time their children have grown up (136). To date, however, comprehensive progress is still needed to make testicular tissue cryopreservation clinically applicable.

Testicular stem cells (TSC) can be stored in immature testicular tissue or a cell suspension. Detailed procedures of both strategies, including sample preparation, storage containers, cryoprotection, and cooling and warming process, have been elaborated elsewhere (137). Different cryopreservation strategies, including slow freezing and vitrification, have been attempted in human and animal models, leading to conflicting results (138–140). But slow freezing remains the most popular option for testicular tissue cryopreservation (130, 131), with both controlled (141–143) and uncontrolled (138) slow-freezing protocols under use.

The overall process of immature testicular tissue cryopreservation and fertility restoration procedures have been vividly described in a recent review (144). Potential methods for fertility restoration include autologous graft of immature testicular tissue (145), injection of testicular stem cells into the testis (146, 147), and *in vitro* maturation of TSCs (148, 149). The main advantage of ITT graft is the preservation of TSCs within their original niche (130). The maintenance of cell interaction and paracrine are preferable for tissue maturation, stem cell self-renewal, and differentiation (50, 150, 151). However, several male pediatric cancers, including testicular cancer, leukemia, and lymphoma, are prone to metastasize to the testes (152), significantly increasing the risks of malignancy relapse after autograft (33, 142). *In vitro* maturation of TSCs and reinjection of a TSC suspension free of malignant cells into testes, by contrast, can avoid the risks of cancer reoccurrence (50). But the original supporting conditions for *in vivo* spermatogenesis are absent.

## Clinical outcomes after fertility preservation

### Oocyte cryopreservation

For female patients, embryo preservation, if available, is the best method for female fertility preservation. However, lack of a permanent partner and ethical concerns to use of donor sperm make oocyte cryopreservation adopted far more frequently in post-pubertal adolescent patients (17). Poorer outcomes are seen compared to embryo cryopreservation due to oocyte degeneration after thawing (53), which is even greater when it comes to immature oocyte cryopreservation (153–155).

The clinical outcomes using cryopreserved mature oocytes have been steadily improving as freezing/thawing techniques evolve and ICSI is used for fertilization (63). Some randomized control trials compared the clinical outcomes between vitrified oocytes and fresh oocytes, which confirmed the non-inferiority of vitrified oocytes to fresh oocytes in terms of fertilization rate, embryo development, implantation rate, clinical pregnancy rate, and live birth rate (62, 156), with similar conclusions drawn in other publications (61, 66–69, 157). The fertilization rates of oocytes with ICSI after thawing based on a large sample size ranged between 70.0% and 81.6% (61, 67, 158). The implantation rates fluctuated around 40% per embryo transferred (62, 67, 68). Notably, some studies found that the implantation rate using autologous vitrified oocytes was significantly lower than that of donor oocytes (63, 157). Current data suggests the clinical pregnancy rates per transfer can be as high as 50.7% to 62.6% (61, 63, 157, 159) whereas live birth rates per transfer range between 39% and 52% (61, 67, 157). A study found poor success rates among cancer patients than those who pursue elective oocyte preservation, but no statistically significant differences were observed after correction for age and controlled ovarian stimulation protocols (159). Current data collectively suggest that oocyte cryopreservation is an effective method for female fertility preservation. However, the efficiency of frozen/thawed oocytes remains unknown, which is vital for appropriate consultation regarding the number of oocytes to freeze to obtain at least a live birth in the future (61). Preliminary investigations revealed the overall percentage of warmed mature oocytes resulting in a live birth ranged between 4.2% and 10.8% (9.3 to 23.8 vitrified/thawed oocytes can lead to a live birth) (61, 63, 66). Besides, most previous studies were based on adult women aged >30 years, concluding that advanced age was negatively correlated with reproductive outcomes (61, 160, 161) and warranting more studies to counsel adolescent patients on the ideal number of oocytes needed to achieve a live birth (162).

While evidence indicates that advanced paternal age is less associated with increased rates of human embryonic aneuploidy (163, 164), it is well known that maternal age is highly correlated with oocyte/embryo aneuploidy (165) and it is one of the strongest predictors of IVF success (166). Interestingly, a recent study revealed that aneuploidy is also common among very young women (167). Gruhn et al. investigated the oocyte aneuploidy rates in women aged 9 to 43 years and found that oocytes aneuploidy rates from young women aged under 20 were significantly higher than those from women in their 20s and early 30s, which exhibited a U-shape curve. In comparison to women in their 20s to early 30s, younger women are also reported to experience higher rates of embryonic aneuploidy (165) and miscarriage (168), which deserves attention when providing counselling to post-pubertal adolescent patients on the clinical outcomes of oocyte cryopreservation.

Oocyte cryopreservation is an effective method for female fertility preservation. However, the relationship between long-term freezing and clinical efficacy, or offspring safety requires ongoing study (169). A multicenter study assessed the outcomes of oocytes cryopreserved for up to 48 months, no apparent differences in post-thawing oocyte survival, fertilization, cleavage, implantation, and live birth were observed when compared with those preserved for shorter periods (170). A more recent study reported a woman whose oocyte was frozen for 14 years and resulted in a healthy baby after fertilization with ICSI (171). On the other hand, congenital malformations were reported at rates ranging from 0.005% to 5.6% in several large cohorts (172, 173), which is close to the incidence in the USA national birth record in 2019 (3%) (169). Nonetheless, long-term follow-up of these children based on large cohorts is still needed.

### Ovarian tissue cryopreservation

Currently, ovarian tissue cryopreservation is already considered an accepted technique for female fertility preservation given its success in restoring ovarian function and fertility (53). Recent studies demonstrated that reimplantation of ovarian tissue in the pelvic cavity resulted in the restoration of ovarian function in 85% to 95% of adult recipients (101, 103, 113, 174), as evidenced by the return of menstruation (98, 113, 175) or pregnancy (102, 113). Researchers also examined the serum hormone profiles before and after ovarian tissue transplantations, demonstrating a gradual decline in both follicle-stimulating hormone (FSH) and luteinizing hormone (LH) levels and return to premenopausal levels 4 to 5 months after transplantation, which was accompanied by the resumption of menstrual cycles and the disappearance of menopausal symptoms (45, 99, 176, 177). However, restoration of ovarian endocrine function may not be reflected by AMH, which was almost undetectable in most cases (176, 178, 179), indicating a limited follicular population in the graft. According to Diaz-Garcia et al., the mean intervals between ovarian tissue transplantation and ovarian function resumption was 94.3 days (103), with most reported cases ranging between 3 and 6.5 months (98, 99, 104, 108, 123, 124, 176, 178, 180–182). The time frame of ovarian function resumption is consistent with that of folliculogenesis (183).

Duration of ovarian function after grafting can depend on the quantity of primordial follicles at the time of transplant and proportion that survive the grafting process (99). The mean duration is approximately 4 to 5 years in humans (174). However, in a study including 41 young women (aged 32.9 on average at the time of OTT), more than half of the transplantations resulted in a functional life span between 1 and 4 years, with some cases lasting for more than 10 years while several cases lasting less than one year (99). The longest duration of restored ovarian function recorded to date is 13.5 years by repeating the transplantation procedure (98). The heterogeneity indicates the necessity of improving and standardizing procedures for ovarian tissue cryopreservation and transplantation.

Since the first live birth report after ovarian tissue autografting in 2004 (96) and the second in 2005 (97), the number of pregnancies and live birth have continued to climb steadily, showing an exponential trend (82). Live births after autografting of ovarian tissue cryopreserved before (184, 185) and after (186, 187) menarche have been reported recently. The number of live births after ovarian tissue transplantation was estimated to exceed 200 in 2020 (188). However, the total number of transplantations worldwide (the denominator) is unknown, leading to the unavailability of accurate pregnancy rates and live birth rates. In an early study based on five centers worldwide including 111 patients, the pregnancy rate and live birth rate were 29% and 21%, respectively (105). These figures were subsequently confirmed by several case series and pooled analyses with larger sample sizes (98, 99, 101–104, 113, 182), yielding a clinical pregnancy rate between 27.3% and 65.6%, and a live birth rate between 18.2% and 43.3%, respectively. Nonetheless, these results have been confounded by both patient factors (i.e. age at OTC/OTT, exposure to gonadotoxic therapy, the number and size of ovarian slices replaced, and residual ovarian function, *etc.*) and technical factors (i.e. surgical techniques, application of proangiogenic agents, the assistance of artificial reproductive techniques, *etc.*) (100).

Currently, most studies focus on adult subjects with little attention to ovarian tissue cryopreservation and transplantation from tissue taken from prepubertal children and post-pubertal adolescents. There are several large cohorts of young girls reporting ovarian tissue cryopreservation, but the return-to-use rates are extremely low (45, 189, 190), leaving limited data to evaluate the endocrinological and reproductive function after ovarian tissue replantation in this population. **Table 2** includes some of the current reports on ovarian tissue transplantation that were cryopreserved at the age of ≤ 20 years. In 2012, there were two cases of ovarian tissue transplantation in pre-pubertal girls to induce puberty (191, 192), resulting in gonadotropins decline and estradiol secretion. Although ovarian activity ceased about 2 years after the grafting, both patients established normal menstrual cycles and secondary sex characteristics shortly after the grafting, demonstrating proof of concept in inducing puberty. As the amount of tissue required for pregnancy and parenthood is unknown in any individual, concerns regarding use of OTT for pubertal induction remain (198, 199).

**Table 2:** Current information about ovarian tissue cryopreservation and transplantation in prepubertal children and post-pubertal adolescents.

Reference	Age at OTC	Age at OTT	Hormonal restoration	Interval between graft and restoration	Duration of ovarian function	Pregnancy	Live birth	Notes for results
Ernst et al. 2013 (191)	9	13.5	YES	4 Mon	19 Mon	NA	NA	NA
Matthews et al. 2018 (185)	9	23	NA	NA	NA	YES	YES	IVF
*Poirot* et al. 2012 (192)	10	13	YES	2 Mon	2 years	NA	NA	NA
Demeestere et al. 2015 (184)	14	24	YES	4.5 Mon	NA	YES	YES	NC
Meirow et al. 2016 (107)	14	21	NO	–	–	NO	NO	Graft failure
	19	27	YES	NA	4 Mon	NO	NO	IVF failure
	19	31	YES	NA	NA	YES	NO	Ongoing pregnancy
	19	37	YES	NA	NA	NO	NO	IVF failure
Donnez et al. 2011 (193)	17	24	YES	3.5 Mon	NA	YES	YES	NC
	20	23	YES	4 Mon	NA	YES	YES	NC
	20	NA	YES	3.5 Mon	>8 Mon	YES	YES	NC
Póvoa et al. 2016 (194)	18	28	YES	1 week	>6 Mon	NO	NO	Embryo cryopreserved
Donnez et al. 2012 (186)	18	28	YES	24 weeks	NA	YES	YES	IVF
Rosendahl et al. 2011 (122)	NA	19	YES	NA	>18 Mon	NA	NA	Embryo transfer
Revel et al. 2011 (187)	19	23	YES	NA	>9 Mon	YES	YES	IVF
Schmidt et al. 2011 (176)	19	NA	YES	16 weeks	>18 Mon	NO	NO	IVF failure
Roux et al. 2010 (195)	20	23	YES	9 weeks	NA	YES	YES	NC
Van der Ven et al. 2016 (196)	20	27	YES	NA	>1 year	YES	YES	NC
	20	29	YES	NA	>1 year	YES	NO	NC, Tubal pregnancy
Callejo et al. 2013 (197)	20	30	YES	4.5 Mon	NA	YES	YES	IVF

OTC, ovarian tissue cryopreservation; OTT ovarian tissue transplantation; NC, natural conception; IVF, *in vitro* fertilization

Another subject of study is the relationship between follicular density/quantity and the longevity of restored ovarian function. Several studies observed an association between younger age at the time of ovarian tissue cryopreservation and preferable clinical outcomes including the longevity of graft survival (200) and live birth rates (102, 103, 113, 196), though challenged by another study (99). The association may be, at least partly, explained by the greater number of follicles residing in the tissue upon harvest in the younger population. In fact, the primordial follicle pool in cryopreserved ovarian tissue retrieved from prepuberty adolescents is significantly larger than those from older patients (189, 201). However, in the report by Ernst et al. (191) and Poirot et al. (192), the ovarian tissue was cryopreserved at the age of 9 and 10 years, respectively. After the graft, however, the endocrinological function was maintained for merely 19 months and 2 years, respectively, much shorter than the average duration reported in adult subjects. These discrepancies mean much optimization is still needed to maximize the clinical outcomes.

Notably, some patients may undergo chemotherapy before ovarian tissue cryopreservation. The latest studies have demonstrated similar resumption in ovarian function and pregnancy rates per woman in patients who received low gonadotoxic risk chemotherapy compared to those who were chemo-naïve (98, 104, 182). Importantly, ovarian harvest in those who have achieved complete remission following chemotherapy may reduce the chance of malignant contamination among patients with leukemia (117–119). However, chemotherapy containing alkylating agents does adversely impact the clinical outcomes including both the pregnancy rate and live birth rate (98).

### Sperm and testicular tissue cryopreservation

Sperm cryopreservation is the most successful method for male fertility preservation. While some studies suggested reduced sperm viability and motility after thawing (202, 203), cryopreserved sperm from patients with a previous malignancy has comparable potential to obtain a clinical pregnancy as ART evolves, especially with the use of ICSI for fertilization (204, 205).

The documented clinical pregnancy rates using thawed sperm collected before cancer therapy ranges from 18% to 57% (204, 206–210). Meanwhile, a higher success rate is observed in ICSI programs, followed by IVF, with intrauterine insemination (IUI) being the least successful (206–208, 210). According to an early study, it took a median of 3 cycles to get pregnant in ICSI, whereas 8 cycles were required in IVF (211). Similar pregnancy rates have been observed compared with non-cancer control or fresh sperm (205, 208). A large cohort involving 272 males with cancers reported a live birth rate of 62.1% per patient, comparable to the non-cancer infertile population (205). Specifically, the outcomes of couples using testicular sperm do not differ between fresh and frozen-thawed sperm among patients with Klinefelter syndrome (212, 213), obstructive azoospermia (214), and non-obstructive azoospermia (215, 216). But the cumulative live birth rate was lower than that of ejaculated sperm (216, 217).

Sperm can be stored for decades under ultra-low temperatures. The longest duration of sperm cryopreservation to date is 28 years, which successfully resulted in a healthy live birth with IUI (218). Unfortunately, it remains unknown whether the freezing-thawing process poses an adverse impact on the long-term development of children born with cryopreserved sperm.

For those patients who had their testicular tissue cryopreserved, the fertility restoration strategy includes autologous grafting of immature testicular tissue (145), injection of TSCs into the testis (146, 147) and *in vitro* maturation of TSCs (148, 149) and these options have been extensively discussed in a recent review (130). Due to its experimental nature, clinical outcomes on testicular tissue transplantation in human subjects are still unavailable regardless of great achievements obtained using animal models (20, 33, 50).

Transplantation of immature testicular tissue appears to be one of the most promising methods for male fertility preservation. Since the live birth of mice and rabbits after fresh and cryopreserved immature testicular tissue transplantation in 2002 (219). Achievements have been made on various animal models, with promise toward clinical use. In summary, functional spermatogenesis after testicular tissue graft has been made possible in mice (220), ferret (221), sheep (222), pigs (223), collared peccary (149), bison (224), buffalo (225), Coturnix japonica (226), and in non-human primates including marmoset (227), cynomolgus monkey (228), and rhesus macaques (145, 229). In some species, offspring using graft-derived sperm with ICSI have been reported (145, 223, 226, 228), proving its potential in fertility preservation and restoration in prepubertal males. The anatomy and physiology of the testis in non-human primates resemble humans the most and make them perfect preclinical models for ITT transplantation research (130). Most recently, fresh and cryopreserved testicular tissues from prepubertal rhesus macaques were autologously transplanted under the back skin and scrotal skin after castration. Surprisingly, all grafts survived, grew, and restored testosterone reproduction as well as endogenous spermatogenesis. A healthy female baby was produced with graft-originated sperm (145). This study marks the biggest milestone for testicular tissue cryopreservation and auto-transplantation toward clinical translation.

Reinjection of TSCs was first introduced in 1994 (146, 147). TSCs isolated from immature mice were injected into the testes of infertile hosts and successfully colonized the seminiferous tubules. The host mice restored natural spermatogenesis and produced offspring using sperm from donor tissue. From that onwards, the method has been proven successful in multiple animals. Offspring were obtained by either natural mating or assisted reproductive techniques after fertility recovery in mice (230, 231), rats (232, 233), goats (234), sheep (235), chicken (236), and zebrafish (237). However, the overall success using primate models is limited. *In vivo* spermatogenesis was recovered (229, 238, 239), and in some cases, embryo formation was documented (238), but no offspring have been reported yet.

*In vitro* maturation of TSCs was also established on mice models which successfully resulted in offspring using round spermatids with ICSI (230). Researches focusing on rats (240), pigs (241), calf (242), and buffalo (243) has successfully induced post-miotic cells (haploid germ cells), and some studies progressed to the formation of preimplantation blastocysts (244). But healthy offspring was reported in mice exclusively (130). IVM of TSCs in non-human primates (245, 246) and humans (247–249) has led to similar results. Only post-meiotic cells were documented. Overall, this technique is still in its infancy.

## Transgender population

Transgender individuals represent a special population who recognize internal gender as different from biological gender. The latest statistics estimated that there are 150,000 young and 1.4 million adult transgender women (transwomen, MtF) or transgender men (transmen, FtM) in the United States (250). In addition, there is a trend towards presentation at younger ages (251). To alleviate gender dysphoria, many of them choose gender-affirming therapy including gender-affirming hormone therapy (GAHT) and gender-affirming surgery (GAS) (43), rendering temporary subfertility or permanent sterility (252). Accordingly, gender-affirming therapy, both hormonal and surgical, is one of the indications for fertility preservation (43, 253).

A variety of studies suggest transgender individuals have a strong desire for parenthood. 62% to 82% of transgender individuals want to have children, biological or adopted (254–256). But the desire to have children declines throughout the GAHT process (257). Meanwhile, nearly half of the transgender adolescents noted that their desire to have their biological children may change when they grow up (258) whereas a proportion of them regretted not undergoing fertility preservation (259, 260). A recent survey revealed that almost all (94.6%, 387/409) transgender respondents agreed that fertility preservation should be offered to all transgender individuals (261).

Several scientific societies have issued guidelines navigating health care to the transgender population, recommending that all transgender individuals should receive a consultation about potential fertility risks of gender-affirming treatments and preservation options before transition (43, 253, 262). However, no guideline specifies the optimal time to initiate discussion and counseling, leading to some situations where patients have their first discussion about fertility preservation after the initiation of gender-affirming therapy (263, 264). Inadequate and belated information provision puts patients in a dilemma between fertility preservation and discontinuation/delay of gender-affirming therapy. Considering most transgender persons are reluctant to postpone or suspend gender-affirming therapy (254, 261), it appears even more important to start consultation as earlier as possible.

### Fertility preservation for transmen

Oocyte cryopreservation, embryo preservation, and ovarian tissue cryopreservation are established methods for fertility preservation for transmen. While ovarian stimulation and the accompanying unpleasant experience of estradiol elevation, vaginal examination and oocyte retrieval are unavoidable for oocyte cryopreservation (265, 266), ovarian tissue can be obtained at the time of gender-affirming surgery. For transmen who have not started GAHT, ovarian stimulation protocols are the same as those used for infertility (251). GnRH antagonist protocol can be considered for its efficacy in oocyte yield (32), and the addition of letrozole can reduce estradiol levels and related symptoms (267).

Some transmen may have already started testosterone treatment before ovarian stimulation. It was previously deemed that testosterone induced polycystic ovary syndrome (PCOS) (268). But recent studies demonstrated testosterone exposure for more than a year did not disturb ovarian follicle distribution (269, 270). Some studies suggest temporary discontinuation of hormonal therapy before ovarian stimulation (251, 265). While some small studies demonstrated no differences in oocyte yield between transmen with continuous hormonal therapy and ciswomen (271–273).

*In vitro* maturation of immature oocytes from oophorectomy can be another source of oocytes (274). However, a recent study demonstrated the low feasibility of this strategy for fertility preservation (275). Almost 2,000 cumulus-oocyte-complex (COCs) were collected at the time of oophorectomy and merely 23.8% of them matured after *in vitro* culture. Of the 151 out of 208 mature oocytes that survived vitrification/thawing, 139 oocytes were fertilized with ICSI, leading to 48 normal fertilizations (34.5%) and 4 transferable blastocysts. Collectively, given the poor maturation rate (28% to 36%) and utilization efficacy after IVM (59, 276), as well as lower pregnancy rates and higher pregnancy loss rates (277–279), IVM should not be used as the only method for fertility preservation in transmen (280).

For prepuberty transmen and those who are unwilling to accept ovarian stimulation, ovarian tissue cryopreservation is the sole option for fertility preservation (253). Ovarian tissue can be obtained after oophorectomy without testosterone discontinuation (280). Auto-transplantation of cryopreserved ovarian tissue has resulted in more than 200 live birth (188). But there is no report of ovarian tissue transplantation in transmen.

### Fertility preservation for transwomen

Sperm cryopreservation is a reliable option for fertility preservation among transwomen (253). Sperm may be obtained by either masturbation, assisted ejaculation, or TESE (33, 129). Cryopreserved sperm could be used for IUI, alternatively, IVF/ICSI using oocytes from a donor or cisgender female partner (251). Cumulative live birth rate using frozen/thawed sperm before anticancer treatment can be as high as 62.1% with ART (205), but these results may not apply to the transgender population because gender-affirming therapy (280–282) and some behavioral factors (283, 284) pose reversible or irreversible threats on semen quality.

For prepubertal transgender girls, testicular tissue cryopreservation remains the only option for fertility preservation (251). Some transgender girls may have received puberty suppression and estrogen supplementation at different pubertal stages (43), leading to decreased testosterone levels. The deficiency of intratesticular testosterone results in severe spermatogenesis dysfunction (280). The effect of testosterone suppression on spermatogenesis in adults have been extensively investigated in cisgender male contraceptive research and is reversible after cessation of suppression therapy (285). But the effect on pubertal transgender girls remains partly unanswered. de Nie et al. investigated the histology of testes using orchiectomy samples under testosterone suppression and/or estrogen exposure (286). They found only immature germ cells (spermatocytes and spermatogonia) present in the seminiferous tubules when medical intervention started at Tanner stage 2-3, with additional mature sperm observed in 57% of subjects who initiated medical treatment at Tanner stage 4 or later. These findings indicate the potential of testicular tissue cryopreservation for fertility after the initiation of puberty suppression and estrogen therapy. However, testicular tissue cryopreservation is currently experimental (131). Future use depends on advances in IVM of testicular stem cells since the *in vivo* microenvironment for spermatogenesis is unobtainable after orchiectomy.

## Fertility preservation program

### Current challenges

Several scientific societies have issued clinical practice guidelines for fertility preservation in pediatric and adolescent cancer populations (11, 17, 32, 50, 76), but surveys indicate limited knowledge of guidelines and poor compliance with recommendations by medical professionals (12, 13, 287, 288). According to a survey in the United States, only 46% and 12% of oncologists routinely refer male and female pubertal patients to fertility preservation services before cancer treatment, respectively (289). Similar research among adolescent and young adult cancer survivors reveals that 80% and 68% of male patients can recall being offered information about potential fertility impairment and referral to fertility preservation service, but the figures for female patients are only 48% and 14%, respectively (290). Evidence suggests that most patients and their parents are dissatisfied with the content of information that healthcare professionals provided concerning fertility risk and available options to preserve it (17). Younger patients and their parents are concerned about fertility issues, but they find it difficult to extend discussions with their physicians (291–293). A major barrier hindering preferable fertility preservation practice is the lack of a structured and coordinated fertility preservation program (12, 13). Meanwhile, some well-organized fertility preservation programs have been proven very successful (294–297).

### Education for health professionals

Clinicians’ knowledge and attitudes toward fertility preservation significantly influences fertility preservation practice (12, 13, 288, 298). Physicians may be restrained by their limited understanding of the gonadotoxic nature of chemotherapy or radiotherapy, potential risks regarding future family planning patients may face, fertility preservation possibilities, and the highly time-sensitive nature of this intervention (12, 288, 299). While some physicians have realized the sensitivity and importance of fertility issues, oncologists tend to provide treatments that maximize the chance of survival and regard fertility issues as a non-priority (12, 76). However, based on the increasing proportion of children and adolescents who survive malignancies (1–3), it is crucial to focus on patients’ quality of life (294, 300) and incorporate fertility preservation into cancer care (76, 301). Accomplishing the objectives should start with educating healthcare professionals (12, 76, 295), possibly including pediatric oncologists, radiation oncologists, gynecologists, urologists, hematologists, surgeons, nurses (11, 32, 127, 302). The clinical team is supposed to have sound knowledge about infertility risk assessment and fertility preservation consultation (11, 12, 17, 32, 76) as fertility risk assessment of specific agents or therapy regimes has been extensively discussed in previous guidelines (25, 47–50). Some of the most common gonadotoxic agents are presented in **Table 3**. To better facilitate fertility preservation, it is suggested to be incorporated into general education of oncology (76, 294, 302). According to a recent study, a simple fertility training program can considerably increase oncologists’ knowledge of infertility risk assessment and fertility preservation strategies (303).

**Table 3:** Estimated risk of chemotherapy for gonadal function (46).

High risk	Medium risk	Low risk
Cyclophosphamide	Cisplatin	Vincristine
Ifosfamide	Carboplatin	Methotrexate
Chlormethine	Doxorubicin	Dactinomycin
Busulfan		Bleomycin
Melphalan		Mercaptopurine
Procarbazine		Vinblastine
Chlorambucil		

### Informed consent

Discussion about potential fertility risks and methods to preserve it must begin at the time of diagnosis (mostly at the time of cancer diagnosis) (11, 76). It may be necessary to assume that every young patient diagnosed with a disease that requires gonadotoxic therapy is concerned with fertility issues (127). One approach to promote fertility preservation practice would be to make it the clinicians’ (pediatricians and oncologists in particular) regular responsibility to evaluate potential fertility risks and initiate discussions about fertility preservation including options, benefits, risks, and costs with patients (53, 294). It is proposed that the clinical team assign a knowledgeable team member to offer a comprehensive and in-depth consultation, providing detailed information concerning potential risks of fertility loss with proposed treatment regimes, current options for fertility preservation, possible risks of delaying treatment, the overall prognosis of cancer, and location of local or regional fertility preservation service, and be ready to answer questions that patients are interested (294).

A formal and separate discussion about fertility issues is advocated to ensure patients’ full comprehension of potential fertility risks and fertility preservation options (136, 294). Importantly, considering the sensitive nature of the age group and topics, patients should be offered a chance to speak freely without the presence of their parents (136, 294). In addition, institutions can design printed or online education materials for interested patients and the ordinary public to facilitate information provision (32). Notably, both formal conversation and supplementary materials should be organized in lay languages and avoid professional terms to ensure that recipients can comprehend the information (127, 294). The provision of relevant medical information should be documented in the patient’s medical record and patients who decide to seek fertility preservation must provide written informed consent (32, 294).

### Referral

The establishment of a standardized intra-institutional or inter-institutional referral pathway between the clinical team and fertility preservation team is strongly suggested (32, 53). In addition, a coordinator between both teams would play a crucial role in navigating the fertility preservation process (32, 127). This institutional arrangement has several advantages.

Firstly, it facilitates fertility preservation consultation (32, 294, 304). Concerns by oncologists about lack of efficacy can hinder referral of patients to fertility preservation services (12), thus, collaboration with fertility specialists allows the chance to eliminate their doubts and update them on the latest fertility preservation technologies (76). Furthermore, fertility preservation specialists can contribute to fertility education provided to oncologists as described above.

Secondly, it facilitates referral (32, 294). Another barrier hindering oncologists’ raising fertility discussion is that they are not aware of local or regional fertility services (12, 302). A direct link between the clinical team and the fertility preservation team would greatly facilitate referral. For instance, a male patient who wishes to bank sperm can make first contact with the sperm bank (294, 296, 304). This would not only save patients time and costs upon the life-changing event (cancer) but also reduce their psychological stress.

Thirdly, it enables optimal decision-making (76). The fertility team is proficient in fertility preservation techniques. But they must cooperate with the clinical team to decide whether it is medically feasible to perform fertility preservation procedures and determine the optimal timing and strategy (53, 76, 304).

Finally, it reduces repetitive work and improve efficiency. As described above, a fertility specialist can engage in both patient counseling and proposing a feasible fertility preservation plan with the clinical team, saving their time and energy. A fertility specialist can focus on other affairs concerning fertility preservation, such as logistics of the procedure, cryopreservation of relevant materials, and future use. Considering the highly time-sensitive nature of fertility preservation, any improvement in efficiency would benefit patients significantly.

### Effectiveness and efficiency

Based on previous literature, there are two identified key factors in establishing an effective and efficient fertility preservation program, namely sensitive faculties (294, 297, 304, 305) and a rapid referral pathway (294, 295, 304). Those patients who require fertility preservation do not generally visit a reproductive clinic. Instead, most of these patients are identified in a pediatric or an oncological clinic. The optimal strategy would be educating pediatricians, oncologists, and relevant nurses to raise their sensitivity to fertility issues (32). More importantly, they should be familiar with common gonadotoxic therapy so that they can assess fertility risks and decide whether to refer a patient or not and fertility preservation can be moved forward quickly. Moreover, a rapid referral approach would substantially increase patients’ awareness and accessibility to fertility preservation services. Patients and their families tend to feel overwhelmed upon the diagnosis, sparing them limited time and energy to access information concerning fertility risks and ways to preserve it (294). As mentioned previously, even some medical professionals lack the necessary knowledge about the potential fertility risks of chemotherapy and radiotherapy and available local or regional fertility preservation services (12, 13, 302). It is reasonable to assume most patients and their families are ignorant of these risks and relevant services. A rapid referral pathway significantly reduces the time and costs patients need to access fertility preservation services. The role of the fertility preservation team is relatively dependent on referral since patients do not generally visit them. However, once engaged, they can work with the clinical team and propose an optimal fertility preservation scheme as soon as possible. Given the urgency of both cancer therapy and fertility preservation, any delay in the process would potentially harm patients’ interests.

## Future directions

Although different communities have made great achievements in fertility preservation, there are still some restrictions in clinical practice. For instance, unlike embryo or gamete cryopreservation, transplantation of cryopreserved ovarian tissue generally leads to variable outcomes between patients and institutions, warranting optimization and standardization of this technique. Currently, the greatest challenge is the massive follicle atresia after transplantation, with more than 70% of primordial follicles failing to survive (116). Future studies should focus on promoting follicular survival by enhancing revascularization and reducing ischemia-reperfusion injury soon after the transplantation (54). Potential strategies include transplanting ovarian tissue with biocompatible decellularized extracellular matrix scaffold (306), and the utilization of antioxidants, anti-apoptotic agents (307, 308), or proangiogenic factors (309, 310). In addition, artificial ovary (311) and complete *in vitro* development of follicles (312) are potential strategies for female fertility restoration. Recent advances in testicular tissue autograft from prepubertal rhesus macaques are encouraging (145), highlighting its promise in male fertility restoration but more similar studies are required to move it towards open clinical trials. Meanwhile, additional efforts are required to address the risks of malignancy reintroducing and investigate the long-term health of children born through fertility preservation programs. Finally, disciplines must collaborate to set up an effective and efficient fertility preservation program that enhances awareness among medical professionals and patients and removes the barriers to fertility preservation services.

## Conclusions

In conclusion, fertility preservation is an increasingly important issue in pediatric and adolescent healthcare as most malignant diseases are curable with contemporary means. Mature gamete cryopreservation is the most reliable and successful strategy for fertility preservation when embryo freezing is not practicable. In addition, ovarian tissue cryopreservation is an effective option for female fertility preservation though there is room for improvement in its efficacy. Autograft of immature testicular tissue is currently the most promising method for prepubertal patients, but additional efforts are required to gather data on animal models and in clinical trials.

## Author contributions

LC, ZD and XC: Substantial contribution to the conception and design of the work. LC: Participation in the acquisition of literature. LC and XC: Manuscript drafting and revision. All authors contributed to the article and approved the submitted version.
